# Validation of the Brazilian Healthy Eating Index-Revised Using Biomarkers in Children and Adolescents

**DOI:** 10.3390/nu10020154

**Published:** 2018-01-30

**Authors:** Roseli B. D. Toffano, Elaine Hillesheim, Mariana G. Mathias, Carolina A. Coelho-Landell, Roberta G. Salomão, Maria O. R. V. Almada, Joyce M. Camarneiro, Tamiris T. Barros, José S. Camelo-Junior, Serge Rezzi, Laurence Goulet, Maria P. Giner, Laeticia Da Silva, Francois-Pierre Martin, Ivan Montoliu, Sofia Moco, Sebastiano Collino, Jim Kaput, Jacqueline P. Monteiro

**Affiliations:** 1 Department of Pediatrics, Ribeirão Preto Medical School, University of São Paulo, Bandeirantes Avenue, 3900, Ribeirão Preto 14049-900, Brazil; elainehillesheim@usp.br (E.H.); marimathias@hotmail.com (M.G.M.); k_rolacoelho@hotmail.com (C.A.C.-L.); rsalomao@usp.br (R.G.S.); madovale@usp.br (M.O.R.V.A.); joycecamarneiro@gmail.com (J.M.C.); tamiris.barros.7@gmail.com (T.T.B.); jscamelo@fmrp.usp.br (J.S.C.-J.); jacque@fmrp.usp.br (J.P.M.); 2 Nestlé Institute of Health Sciences, Innovation Square, École Polytechnique Fédérale de Lausanne, Campus, 1015 Lausanne, Switzerland; serge.rezzi@rd.nestle.com (S.R.); laurence.goulet@rd.nestle.com (L.G.); mariapilar.giner@rd.nestle.com (M.P.G.); laeticia.dasilva@hotmail.com (L.D.S.); francois-pierre.martin@rd.nestle.com (F.-P.M.); ivan.montoliuroura@rd.nestle.com (I.M.); sofia.moco@rd.nestle.com (S.M.); sebastianocollino@gmail.com (S.C.); jkaput@gmail.com (J.K.)

**Keywords:** Brazilian Healthy Eating Index-Revised, biomarkers, children, adolescents

## Abstract

The Brazilian Healthy Eating Index-Revised (BHEI-R) can be used to determine overall dietary patterns. We assessed the BHEI-R scores in children and adolescents, aged from 9 to 13 years old, and associated its component scores with biomarkers of health and dietary exposure. Three 24-h recalls were used to generate BHEI-R. Biomarkers were analyzed in plasma and red blood cells. Correlation tests, agreement, and covariance analyses were used to associate BHEI-R components with biomarkers. Data from 167 subjects were used. The strongest correlations were between fruits, vegetables and legumes with omega-6 and omega-3 fatty acids, and β-carotene intakes. Milk and dairy correlated with plasma retinol and pyridoxine. All components rich in vegetable and animal protein sources correlated with plasma creatine. Total BHEI-R scores were positively associated with intakes of omega-6, omega-3, fiber and vitamin C, and inversely associated with energy and saturated fat intakes of individuals. Plasma β-carotene and riboflavin biomarkers were positively associated with total BHEI-R. An inadequate food consumption pattern was captured by both biomarkers of health and dietary exposure. BHEI-R was validated for the above dietary components and can be associated with metabolomics and nutritional epidemiological data in future pediatric studies.

## 1. Introduction

Associating individual foods with health outcomes does not account for the chemical complexity of foods and for widely varied eating habits. In addition, intervention studies, using a single nutrient, cannot assess nutrient–nutrient interactions or how combinations of dietary chemicals affect health outcomes. In contrast, diet quality indices can be used to determine overall dietary patterns [1]. The advantage of such indices is that they capture the complexity of human diets in a single value, taking into account population food guides and the interactions between nutrients, food preparation methods, and eating patterns [1,2]. 

In 2004, the Healthy Eating Index (HEI) [3] was adapted to Brazil food culture and subsequently revised in 2011, using the structure of HEI-2005 [4] and the recommendations from the Brazilian Food Guide 2006, with portion sizes for ages above 2 years [5]. This last version is called the Brazilian Healthy Eating Index-Revised (BHEI-R) [2]. The use of the Brazilian guide in the BHEI-R provided a tool to measure and monitor current nutritional recommendations proposed for various stages of life [2]. 

Since biomarkers can more accurately assess the nutritional status for some nutrients, and the errors in its measurements are unrelated to the errors measured by diet quality score methods or self-reported intakes, they provide a better validation of intake. Hann et al. [6] validated the HEI by associating the highest HEI scores with the highest plasma concentrations of α-carotene, β-carotene, β-cryptoxanthin, lutein, and vitamin C. In addition, the highest HEI score was associated with an increased intake of energy, fruits, fiber, folate, and vitamin C, and with a lower intake of total and saturated fat. However, validation of the BHEI-R with biomarkers has not been performed in a pediatric population.

A low concentration of antioxidant factors and other micronutrients has been associated with noncommunicable chronic diseases, such as being overweight, obesity, and metabolic syndrome [7]. Among these micronutrients are retinol [8], carotenoids [8,9,10,11], vitamin E [8], vitamin B6 [11], folate [11], and certain fatty acids, whose insufficiencies or inadequacies are associated with fat deposition, chronic inflammation, high low density lipoprotein (LDL), and insulin resistance [12,13].

The purpose of this study was to assess the BHEI-R as a measure of dietary status in children and adolescents, aged 9 to 13 years, in Ribeirão Preto, São Paulo-Brazil, and to determine the strength of the correlations between BHEI-R scores and plasma concentrations of retinol, β-carotene, riboflavin, pyridoxal, α-tocopherol, 5-methyltetrahydrofolate (5-MTHF), creatine, and red blood cell *S*-adenosyl-homocysteine (SAH), stearic fatty acid, linoleic fatty acid (LA), α-linolenic fatty acid (ALA), arachidonic fatty acid, (ARA), eicosapentaenoic fatty acid (EPA), and docosahexaenoic fatty acid (DHA). If shown to be a valid indicator of nutritional status, this diet intake tool may allow the use of scores to assess the compliance to guidelines which are designed to maintain health and prevent chronic diseases in populations and individuals.

## 2. Materials and Methods

### 2.1. Study Design

Dietary intakes and metabolomics data described in this report were from a crossover N-of-1 intervention study [14]. Nutritional status’s, as measured by metabolites in plasma and red blood cells (RBC), were assessed at baseline (visit 1), after 6 weeks of daily supplementation of vitamins and minerals (visit 2), and after 6 weeks of a washout period (visit 3). To avoid the influence of supplements on plasma metabolite concentrations, this report analyzed metabolomics data only from visit 1. Individuals provided a 24-h dietary recall at each time point, which was used to determine BHEI-R. The mean BHEI-R was calculated from the 24-h dietary recalls at the 3 visits. We used this method since no large differences were found between the 24-h dietary recalls at each of the three visits, which suggested consistency in diets and produced a more reliable assessment of dietary intake. 

### 2.2. Population

Participants in this study were clinically stable children and adolescents, aged 9 to 13 years, and were recruited from three schools on the west side of Ribeirão Preto-SP. This municipality is in the Northeastern region of the state of São Paulo in Brazil. The participants lived in the same neighborhood and had city water, sanitation, electricity, and internet through broadband access. The data collection was performed at the Ribeirão Preto Medical School Hospital, University of São Paulo, Brazil. The study was approved by the internal ethics committee (Process HCRP No. 14255/2010) and by the National Research Ethics Commission (No. 00969412.6 CAAE. 0000.5440). The trial was registered on ClinicalTrials.gov (NCT01823744). The participants were informed about the purpose and procedures of the study and signed a statement of informed consent. Parents of each participant signed informed consent. Participants received Institutional Review Board approved compensation as well as breakfast and lunch following blood draws on each assessment visit. 

At least 50 subjects are necessary for validation studies with biomarkers [15]. Subjects with at least one episode of axillary temperature higher than 37 °C in the 15 days before the blood collection, three or more episodes of liquid stools in the last 24 h, individuals receiving any kind of vitamin or mineral supplement at baseline, or on a supervised diet for reducing weight or on any other type of diet restriction, individuals with a diagnosis of a chronic disease that could interfere with data collection and individuals that had participated in another clinical trial in the previous 4 weeks from the study’s beginning, were excluded from the project. The upper age cut-off was 13 years, 11 months and 29 days at visit 1. Individuals in all weight groups were included. 

### 2.3. Data Collection

All participants submitted to anthropometric, pubertal [16], and economic status assessments [17]. Height and weight were measured after 12 h of fasting, by a dietitian, immediately after blood collection, according to the procedures described by Jellife and the World Health Organization (WHO) [18]. Body mass index (BMI) was calculated and used for nutritional status classification, according to WHO [19]. Usual dietary intakes (mean BHEI-R scores) were assessed by averaging 24-h recalls at baseline (visit 1), and six (visit 2) and 12 (visit 3) weeks. Trained phlebotomists performed blood draws and all blood samples were coded at the time of collection. Blood samples for metabolomic analysis were frozen at −80 °C and analyzed at the Nestlé Institute of Health Sciences, Lausanne, Switzerland. 

### 2.4. Dietary Assessment 

Usual dietary intakes were assessed by three 24-h recalls at baseline (visit 1), and 6 (visit 2) and 12 (visit 3) weeks later. The 24-h recall was done in a stepwise method, adapted [20] to assess a participant’s food intake. One parent or guardian was also present for the assessment. A previously published manual, showing pictures from usual foods in Brazil, in three different sizes (small, medium and large portion size), was used to represent food portions [21]. Diet data were double-checked into the DietWin Professional^®^ software version 2011 (Dietwin Software de Nutrição, Porto Alegre, Brazil), which was used for analyzing dietary intake data. This program includes 5000 food items from six food composition databases (Tabela Brasileira de Composição de Alimentos, Instituto Brasileiro de Geografia e Estatística, U.S. Department of Agriculture, Centro de Endocrinología Experimental y Aplicada, General Directory of Food) and more than 1300 recipes. All foods generated from 24-h recalls were assigned into BHEI-R components, according to the footnote Table 1.

### 2.5. Brazilian Healthy Eating Index-Revised (BHEI-R)

The BHEI-R is estimated by scoring 12 components that characterize different aspects of a healthy diet (Table 1). Each component is evaluated and scored from a minimum of 0 to a maximum of 20. The first 9 components of the BHEI-R are food groups. Total saturated fat, sodium, and SoFAAS (calories from solid fat, alcohol and added sugar) constitute the other 3 components and are scored in the opposite direction to the other components (i.e., lower intakes have higher scores). For all components based on food groups, a full score is given for intakes, at or above, recommended amounts. A zero indicates that no foods in that group were consumed, whereas intermediate numbers of servings are awarded prorated scores. The maximum BHEI-R score is 100 [2]. Since one of the objectives of this study is to correlate the BHEI-R component scores with specific biomarkers, the components that include vegetables were also analyzed without legumes because they are staple foods in Brazil. The components without legumes were not included in the final BHEI-R score. All components were analyzed as continuous variables. The present study used Hann et al. [6] total HEI score categorization to define a “poor diet” as a total BHEI-R score < 65, and a “good diet” as a total BHEI-R score ≥ 85, with scores of 65–74 and 75–84 in between.

### 2.6. Laboratory Analyses

Blood samples were centrifuged to separate red blood cells from plasma. The plasma was frozen at −80 °C. Mass spectroscopy (MS) was used to measure 5-MTHF [22]. Plasma levels of retinol, α-tocopherol and β-carotene were analyzed with ultra-performance liquid chromatography (UPLC) [14]. Plasma riboflavin and pyridoxine were analyzed by reverse phase liquid chromatography (RP-LC) and MS operating in positive electrospray ionization (ESI+) at unit resolution [22]. Red blood cell *S*-adenosyl-l-homocysteine (SAH) was measured by tandem mass spectrometry (LC-MS/MS) [23].

Plasma creatine: The measurements were performed on a Bruker Avance III 600 MHz spectrometer with a 5 mm CPTCI at 300 K (Bruker Biospin, Rheinstetten, Germany). The peak assignment to specific metabolites was achieved using an internal library of compounds in the literature and confirmed by standard two-dimensional nuclear magnetic resonance (NMR) spectroscopy on selected samples. For the statistical analysis, all NMR spectra were converted into 12 K data points over the range of δ 0.4–10.0 and imported into MATLAB software (version 7.14.0 (R2012a; The MathWorks Inc., Natick, MA, USA)), excluding the water residue (water δ = 4.67–4.97).

RBC fatty acids: A 200 μL aliquot of pelleted RBC was added to 200 μL of lysis buffer, mixed 10×, and frozen at −80 °C. The lysis buffer was (1.5 M NH_4_Cl; 120 mM NaHCO_3_ 10 mM EDTA 292.2 mg/mL). Thawed samples were processed, as in Massod et al. [24]. Fatty acids, as methyl ester derivatives, were analyzed by gas–liquid chromatography (GLC), as previously described [25].

### 2.7. Statistical Analyses

Participants who consumed a daily kilocalorie amount lower than 0.79× basal metabolic rate or higher than 2.4× basal metabolic rate (i.e., outliers) were excluded from all analyses [26,27]. All statistical analyses were performed using the Statistical Package for the Social Sciences (SPSS), version 20.0^®^ (IBM, New York, NY, USA). All components were analyzed as continuous variables and presented as median (minimum–maximum). Since biomarkers are biological variables that tend to be non-parametric, Spearman’s rank correlation test was chosen. Correlation analyses were done with Spearman’s test, followed by partial correlation, adjusting for variables such as age, gender, and BMI. True correlations between plasma biomarkers and BHEI-R components were only considered if statistical significance was reached for both crude and adjusted values corrected with a Bonferroni post-hoc analysis. 

Analysis of variance (ANOVA) for repeated measures was used for longitudinal analyses with adjustment for confounding variables. Linear regression covariance analyses (ANCOVA), adjusted for age, sex, BMI, and for energy when needed, were applied to compare the BHEI-R total score groups. Because β-carotene, vitamin E, and retinol plasma concentrations may be influenced by lipoprotein variability and result in extraneous variation in these nutrients, analyses of these variables were adjusted by including total cholesterol (analyzed in the USP Hospital Clinic [14]) in the correlation and ANCOVA analyses with a *p* value < 0.05 as the significance cutoff. All individuals were classified into quartiles according to their component scores and biomarker values. The agreement was assessed through the classification of individuals at the same or at opposite quartiles. 

## 3. Results

A total of 280 children and adolescents met the inclusion and exclusion criteria and were included in the study. After exclusion of under- (*n* = 94) and over-reporters (*n* = 19), 167 subjects were considered for analyses. Seventy-nine individuals were male (47.3%) and 88 were female (52.7%) (*p* > 0.05). The average age was 11.7 ± 1.1 years with 11 individuals (6.6%) in pubertal stage 1, 59 (35.3%) in stage 2, 69 (41.3%) in stage 3, 24 (14.4%) in stage 4, and 4 (2.4%) in stage 5. The majority of the participants belonged to category B2 (37.1%—monthly family income of US$ 812.00) and C1 (28.7%—monthly family income of US$ 494.00) socioeconomic classes. BHEI-R components were similar between pubertal and economic status categories (*p* > 0.05). Based on the classification of BMI for age, two participants (1.1%) were severely thin, 18 (11%) thin, 88 (53%) at the appropriate weight, 32 (19%) overweight, and 27 (16%) were obese. 

### 3.1. Population Food Intake Data

The average total BHEI-R score for all included individuals was 54.8 ± 7.5. One hundred and fifty-two participants (91%) had a total BHEI-R below 65, which is considered a “poor diet”, 15 (9%) were classified in the intermediary category, with scores in between 65–84, and none in the “good diet” category (above 85) [6]. No statistical differences were found in food intake patterns across all the three visits. The worst scores were for whole grains, total vegetables and legumes, dark green and orange vegetables (DGOV) and legumes, total fruits, whole fruits, milk and dairy, and SoFAAS (Table 2). 

Linear regression analyses showed that higher BHEI-R scores were associated with better scores for total grains, whole grains, total vegetables and legumes, DGOV and legumes, total fruits, whole fruits, saturated fat, and SoFAAS. Participants in the intermediate diet intake category, as measured by BHEI-R scores, were eating less energy and saturated fat, and more omega-6 and omega-3 fatty acids, fiber and vitamin C. The higher total BHEI-R scores were associated with higher plasma concentrations of β-carotene and riboflavin, while plasma retinol, SAH, and 5-MTHF approached significance (Table 3).

### 3.2. Correlations Analyses between BHEI-R Components and Biomarkers

The mean of three BHEI-R component scores was evaluated for correlations, with each one of the cited biomarkers using Spearman’s test and data from all participants. Significant correlations were subsequently tested by adjusting for confounding variables, such as age, sex, body mass index, and total cholesterol when appropriate. Table 4 shows the significant results in non-corrected and adjusted correlations. Positive associations were found between whole grains and 5-MTHF. Seven metabolites (LA, ALA, ARA, EPA, DHA, β-carotene, and creatine) were positively correlated with total vegetables and legumes. ALA, retinol, β-carotene, and creatine positively correlated with DGOV and legumes. DGOV, without legumes, positively correlated with DHA, retinol, β-carotene, and SAH. Intake of total fruits positively correlated with LA, ALA, ARA, EPA, DHA and β-carotene. Whole fruits were only positively correlated with β-carotene and riboflavin. Milk and dairy were positively correlated with retinol and pyridoxal. Meat, eggs and legumes were positively correlated with ALA, DHA, and creatine. For almost all the above correlations, the agreement to the same quartile was equal to or above 25%. Negative and significant correlations were found between saturated fat and retinol (*r* = −0.22, *p* = 0.01), and with α-tocopherol (*r* = −0.19, *p* = 0.02). The agreement at opposite quartiles was equal to or above 14% for these associations. The BHEI-R scores for many individuals could not be assigned to quartiles (minimum score for whole grains; maximum score for meat, eggs and legumes) and therefore precluded agreement analyses. After adjusting results obtained for saturated fat with total cholesterol, no correlation was found for retinol (*r* = −0.13, *r* = 0.09) or α-tocopherol (*r* = −0.12, *p* = 0.12). No component was correlated with stearic fatty acid.

## 4. Discussion

The BHEI-R can assess and monitor diet quality based on the current guidelines [28]. The results described here extended the BHEI-R to an analysis of the dietary status’s of Brazilian children and adolescents, aged 9 to 13 years. 

The study sample size was adequate for validation studies with biomarkers [15]. The population was characterized by the same percentage of males and females, with no statistical differences related to the Tanner classification. However, a high prevalence of overweight and obese individuals was found in this cohort, consistent with trends in child and general Brazilian populations [29]. The total BHEI-R score was similar among all economic categories and well below the proposed cut-off of 65 for a qualitatively poor diet [6]. Similar results have also been observed in other Brazilian, European and American studies [30,31,32,33,34,35,36,37,38]. Excessive intakes of sugar and lower intakes of fruits, greenery and vegetables have been previously reported for subjects in other studies conducted in Brazil [29,39].

Low consumption of whole grains, fruits, and DGOV was observed in this population, consistent with other studies in this age group [30,31,35,39]. Only 6.4% of 812 adolescents in a cross-sectional study conducted in São Paulo consumed fruits, vegetables and legumes, according to the Brazilian recommendations [27] and 22% did not consume any type of fruits and vegetables [40]. The results presented here for the milk and dairy component [35,39,41] were consistent with other studies with Brazilian adolescents and may be explained by skipping breakfast [42]. Moreover, beverages with sugar have replaced milk among adolescents [43]. The present study found high scores for the meat, egg and legumes component, corroborating other studies conducted in Brazil [35,39,41], but were higher than intakes described in studies of Europeans [31,44]. These results can be attributed to the high consumption of meat and beans, which are staple foods in Brazil [45].

The sodium component showed an intermediate to low score, indicating high sodium consumption. We observed a higher average score of this component compared to other national studies [35,39,41]. The Brazilian population has high consumption of processed foods that are enriched with sodium [45]. The BHEI-R oil component showed adequate scores and the intermediate score for saturated fat component was similar to the studies reported in Brazil [39,41], but lower when compared to national guidelines [5]. Reduced consumption of this type of fat decreases the risk of dyslipidemia and heart disease and is a consistent public health message [46]. The SoFAAs component score, which includes solid fat, and trans fats, alcohol and added sugar, was very low, indicating a high intake of foods containing these items (with the caveat that alcohol is not typically consumed by this age group in Brazil). Others have found similar results in Brazil [39], which can be partially explained by the high soft drink and stuffed cookie consumption. National data showed that stuffed cookies represented almost 20% of total energy consumed [45]. The above results showed that the cohort of Brazilian children and adolescents, aged 9 to 13 years, need improvement in their diets. Encouraging children and adolescents to improve their dietary patterns may require a reassessment of public and personal health messages.

This study showed moderately strong associations between BHEI-R and plasma and RBC metabolites. Modest to moderate non-adjusted and adjusted correlations were positive and significant between whole grains and 5-MTFH. Whole grains are not good sources of folate, but after the implementation of flour fortification with folic acid in Brazil, serum folate concentrations increased in healthy populations (57% in children and adolescents and 174% in adults) [47]. In addition, others [48] also found positive correlations between a healthier diet containing more whole grains and serum concentrations of folate (*r* = 0.30).

Positive associations between the consumption of total vegetables and legumes, DGOV and legumes and total fruits with AL, ALA, ARA, EPA, and DHA were identified in this population. Recently, Vyncke et al. [49] correlated higher total scores for HEI with healthier diets, suggesting that a higher consumption of vegetables, legumes, and fruits would lead to a better profile of ALA, EPA and DHA. Hence, these metabolites may be good choices for evaluating diet quality. Beans also contribute to dietary intake of these metabolites, since the main fatty acids in the oil fraction from seeds of Phaseolus vulgaris (common bean) are linolenic (34.7–41.5%) and linoleic (30.7–40.3%) [50]. Meat, eggs and legumes were also correlated with DHA. A Brazilian study conducted with adolescents also found that omega-3 polyunsaturated fatty acid intake was positively correlated with the intake of meats and eggs (*r* = 0.39) and beans (*r* = 0.30) [51].

Consumption of total vegetables and legumes, DGOV and legumes, and total and whole fruits had positive correlations with β-carotene. Plasma concentrations of carotenoids are good indicators of vegetable and fruit intake [52]. The total vegetables and legumes and DGOV and legumes components also presented positive correlations with plasma creatine. The participants in this study had a high bean consumption (82.5% ate beans at least once a day, data not shown). When we considered the analyses for DGOV without legumes, the association disappeared, reinforcing the above hypothesis. In addition, the component, meat, eggs and legumes, also presented a positive correlation with creatine. Creatine has been proposed to be a biomarker of animal and vegetable foods rich in protein. However, plasma creatine concentrations are influenced by muscle mass, and hence, its reliability as a precise biomarker needs further clarification [53]. 

Plasma retinol was correlated with DGOV and legumes, which corroborated results of other studies in children and adolescents [54,55]. Riboflavin concentrations were correlated with total and whole fruits, which, to our knowledge, have not been reported by others. However, increased intake of vegetables, fruits, and bread elevated the concentration of riboflavin by 23% in an adult Norwegian population [56]. Further studies are needed to confirm these findings and also the positive association between DGOV and legumes with RBC SAH reported here. 

The milk and dairy component was correlated with retinol and pyridoxal. Milk and dairy products are good source of retinol [57] and the association with pyridoxal concentrations was previously reported [58]. Milk and dairy were among the five top foods contributing to vitamin B6 in a population of Brazilians adolescents [59]. 

The EURopean micronutrient RECommendations Aligned Network of Excellence (EURRECA) developed a scoring system to assess the quality of dietary intake validation studies [60], and our experimental design is classified as having a good quality based on those criteria. Although many of the correlation coefficients found in the present study were modest, they are within the ranges of 0.2 to 0.5 found by others [6,48,51]. Factors contributing to low correlation coefficients include imprecise assessment tools, technical errors in measuring biomarkers, biomarker daily variability, or homeostatic control, genetic background [61], and the possible disparity between the nutrient composition of the current food supply and that reported in food composition databases. Nevertheless, correlation between food intake and biochemical markers provides solid evidence for validating food intake assessments [61]. 

Our study had limitations. Although the participants in this study do not represent all Brazilian children and adolescents because of the small sample size, the local environment, gender, nutritional status, economic and pubertal status were similar between the subjects, limiting the need for external validation [60]. However, further studies must be considered in a larger and more heterogeneous pediatric populations in different geographic, environment, and socioeconomic areas. A second limitation of this study is the fact that 24-h recall used to generate BHEI-R and the plasma biomarkers are more likely to represent recent food intake [62]. This limitation prevents the use of BHEI-R in nutritional epidemiological studies that seek to associate food patterns with chronic diseases, since the kinetics of nutrient absorption and metabolism to plasma or RBC metabolites are difficult to assess. Nevertheless, combining BHEI-R components with biomarkers through the classification of individuals at the same or at opposite quartiles yields more robust results and validation, compared to using either diet or serum data alone [62,63]. In addition, the exclusion of under- and over-reporters also improved results of the classification of individuals within the same or opposite quartiles. The results presented here support the use of nutrient intake assessments, especially for fatty acids, β-carotene, and retinol, which are more sensitive to food intake [64,65]. Despite these limitations, this study is the first to demonstrate the use of BHEI-R assessment tool in a pediatric population and its validation with biomarkers associated with diet quality and healthful food intake patterns. The use of specific food groups and total quality of the diet aids in developing specific, personalized nutrition recommendations.

## 5. Conclusions

Although not nationally representative, the results of this study demonstrated that in the cohort of Brazilian children and adolescents, aged 9 to 13 years, have poor diets, regardless of income level. The negative characteristics of food consumption patterns identified here, such as excessive content of sugar and lower amounts of whole grains, fruits, milk and dairy, and vegetables are consistent with other scientific studies. Although public health agencies utilize results of these type of science studies to develop messaging and policies, inadequate food consumption patterns persist across socioeconomic groups. The levels of metabolite biomarkers reflected these dietary patterns. The strongest correlations were between fruits, vegetables and legumes with omega-6 and omega-3 fatty acids, and with β-carotene. In addition, all components rich in vegetable (legumes) and animal protein sources (meat, eggs) were correlated with creatine. Milk and dairy were correlated with retinol and pyridoxine. The biomarkers positively associated with total BHEI-R score were β-carotene and riboflavin. These results are an important step in the validation of the BHEI-R, emphasizing its potential as a tool for nutrition and health studies in children and adolescents, 9 to 13 years old.

## Figures and Tables

**Table 1:** Distribution of scores and portions for the components of the Brazilian Healthy Eating Index-Revised, 2011.

Components	Score Range				
	0	5	8	10	20
Total grains ^a^	0	2 portions/1000 kcal			
Whole grains ^b^	0	1 portion/1000 kcal			
Total vegetables and legumes ^c^	0	1 portion/1000 kcal			
DGOV and legumes ^d^	0	0.5 portion/1000 kcal			
Total fruits ^e^	0	1 portion/1000 kcal			
Whole fruits ^f^	0	0.5 portion/1000 kcal			
Meat, eggs and legumes ^g^	0			1 portion/1000 kcal	
Milk and dairy ^h^	0			1.5 portion/1000 kcal	
Oils ^i^	0			0.5 portion/1000 kcal	
Saturated fat ^j^	≥15% TEV		10% TEV	≤7% TEV	
Sodium ^k^	≥2.0 g/1000 kcal		1.0 g/1000 kcal	≤0.75 g/1000 kcal	
SoFAAS ^l^	≥35% TEV				≤10% TEV

^a^ All grains plus bread, pasta, cake, pancake and rolls. ^b^ All foods with whole grain cereal, such as brown rice, oats, linseed, whole meal flour and cornmeal. ^c^ All vegetables plus legumes, but only if they surpass the total score for the meat, eggs and legumes component. ^d^ All dark green and yellow vegetables (DGOV) plus legumes, but only if they surpass the total score for the meat, eggs and legumes component. ^e^ All fruits including fruit juices. ^f^ All fruits excluding fruit juices. ^g^ All types of meat, eggs, soy products and all legumes. ^h^ All types of milk excluding pediatric formulas, heavy cream from milk and butter. ^i^ Vegetable and fish liquid oils; non-hydrogenated salad sauce, heavy cream from milk or similar is excluded. ^j^ Total saturated fat from diet. ^k^ Sodium from foods plus sodium from salt. ^l^ Calories from solid fat, alcohol and added sugar. SoFAAS, calories from solid fat, alcohol and added sugar; TEV, total energy value.

**Table 2:** Medians and maximum and minimum values of Brazilian Healthy Eating Index-Revised components in three intake assessments by 24-h recall.

Components *	V1	V2	V3
Total grains	5 (0–5)	5 (1–5)	5 (1–5)
Whole grains	0 (0–5)	0 (0–5)	0 (0–5)
Total vegetables and legumes	2.9 (0–5)	3.1 (0–5)	2.7 (0–5)
DGOV and legumes	4.3 (0–5)	5 (0–5)	3.9 (0–5)
Total fruits	0 (0–5)	0 (0–5)	0 (0–5)
Whole fruits	0 (0–5)	0 (0–5)	0 (0–5)
Milk and dairy	6.5 (0–10)	5.8 (0–10)	7.2 (0–10)
Meat, eggs and legumes	10 (0–10)	10 (0–10)	10 (0.7–10)
Oil	10 (4–10)	10 (4.4–10)	10 (2.9–10)
Saturated fat	8.1 (0.3–10)	8.1 (0–10)	7.2 (0.1–10)
Sodium	8.2 (0–10)	8.5 (0–10)	9.1 (0–10)
SoFAASt	0 (0–20)	0 (0–20)	0 (0–20)
Total BHEI-R	54.3 (31–81)	53.8 (30–89)	53.2 (28–84)

Abbreviations: BHEI-R—Brazilian Healthy Eating Index-Revised; DGOV—dark green and orange vegetables; SoFAAS—calories from solid fat, alcohol and added sugar; V1—baseline time; V2—six weeks from baseline; V3—twelve weeks from baseline. * All values are medians and ranges. *p* > 0.05 for all components in the longitudinal analyses using the general linear model for repeated measures, adjusted for body mass index.

**Table 3:** Brazilian Healthy Eating Index-Revised component scores, nutrient intakes and biomarkers by categories of total Brazilian Healthy Eating Index-Revised.

Variables *	Poor Diet (Score < 65) *n* = 152	Intermediary Category (Score: 65 to 84) *n* = 15	*p* ^†^
*Components*			
Total grains	4.6 (1.9–5.0)	5.0 (4.4–5.0)	<0.001
Whole grains	0.0 (0.00–2.0)	0.0 (0.0–5.0)	0.02
Total vegetables and legumes	2.5 (0.0–5.0)	3.3 (2.2–5.0)	<0.001
DGOV and legumes	3.3 (0.0–5.0)	5.0 (2.7–5.0)	<0.001
Total fruits	1.4 (0.0–4.9)	2.7 (0.0–4.8)	<0.001
Whole fruits	0.1 (0.0–5.0)	2.8 (0.0–5.0)	<0.001
Milk and dairy	6.8 (0.0–10.0)	6.4 (3.1–10.0)	0.86
Meat, eggs and legumes	9.5 (3.5–10.0)	10.0 (7.4–10.0)	0.21
Oil	10.0 (3.8–10.0)	10.0 (9.8–10.0)	0.26
Saturated fat	6.7 (0.6–10.0)	8.5 (6.5–10.0)	<0.001
Sodium	7.7 (0.0–10.0)	8.9 (1.2–9.9)	0.31
SoFAAS	1.3 (0.0–12.0)	8.5 (0.5–18.9)	<0.001
Total BHEI-R	53.2 (34.4–64.2)	67.9 (64.6–83.2)	<0.001
*24-h recall* ^‡^			
Energy, kcal/day	1879 (1256–3313)	1 609 (1270–2080)	<0.001
Carbohydrate, g	244 (140–459)	215 (165–310)	0.25
Lipids, g	68 (26–128)	55 (35–71)	0.09
Cholesterol, mg	215 (46–734)	174 (64–333)	0.27
Omega-6, g	5.98 (0.34–22.99)	7.75 (3.47–16.51)	0.02
Omega-3, g	0.6 (0.1–1.9)	0.8 (0.4–2.1)	<0.001
Saturated fat, g	21.6 (6.7–46.2)	16.3 (8.2–22.8)	0.02
Fiber, g	16.9 (6.1–48.7)	20.7 (14.4–53.8)	<0.001
Protein, g	68.0 (28.39–186.98)	72.5 (41.74–89.55)	0.57
Vitamin A, mcg	552.5 (66.5–10,851)	516.1 (308.9–3568)	0.57
Vitamin E, mg	8.2 (0.3–30.6)	9.3 (4.7–16.6)	0.16
Vitamin C, mg	52.8 (6.0–417.9)	94.1 (32.9–305.7)	0.04
Folate, mcg	137.8 (38.2–690.5)	152.7 (85.5–276.4)	0.18
Vitamin B2, mg	1.8 (0.3–4.4)	1.6 (0.9–2.4)	0.43
Vitamin B6, mg	1.7 (0.4–4.5)	1.5 (0.8–2.3)	0.82
*Biomarkers*			
Retinol, mg/mL ^§^	0.3 (0.2–0.7)	0.4 (0.2–0.6)	0.06
β-carotene, mg/L ^§^	0.1 (0.0–0.7)	0.2 (0.1–0.7)	0.02
Riboflavin, nmol/L	11.5 (5.0–53.5)	11.5 (5.9–68.1)	<0.001
Pyridoxal, nmol/L	7.8 (3.2–17.0)	7.8 (3.1–14.0)	0.16
α-tocopherol, mg/L ^§^	6.0 (2.8–9.4)	6.0 (4.1–11.0)	0.76
5-MTHF mg/dl	19.3 (2.8–77.9)	25.9 (6.8–58.6)	0.08
Creatine, mg/dL	308.2 (201.1–507.7)	299.8 (240.6–426.9)	0.67
SAH, mg/dL	0.8 (0.3–2.0)	0.9 (0.6–2.0)	0.06
Stearic fatty acid, mg/dL	25.9 (16.6–36.9)	25.8 (22.4–30.3)	0.70
LA, mg/dL	16.3 (6.9–28.8)	16.6 (7.3–21.8)	0.74
ALA, mg/dL	0.21 (0.06–0.5)	0.22 (0.1–0.4)	0.38
ARA, mg/dL	20.8 (4.0–38.9)	23.2 (7.7–27.0)	0.55
EPA, mg/dL	0.4 (0.1–1.6)	0.4 (0.2–0.8)	0.85
DHA, mg/dL	3.9 (0.9–10.0)	5.0 (1.9–8.3)	0.26

Abbreviations: 5-MTHF—5 methyltetrahydrofolate; ALA—α-linolenic acid; ARA—arachidonic fatty acid; DGOV—dark green and orange vegetables; DHA—docosahexaenoic fatty acid; EPA—eicosapentaenoic fatty acid; LA—linoleic acid; BHEI-R—Brazilian Healthy Eating Index-Revised; SAH—*S*-adenosyl-homocysteine; SoFAAS—calories from solid fat, alcohol and added sugar. * All results are presented in median (minimum and maximum value). ^†^ *p* value based on linear regression covariance analyses adjusted for age, sex, body mass index (BMI), and ^§^ for plasma cholesterol. ^‡^ All nutrient intakes from 24-h recall were adjusted for energy.

**Table 4:** Crude and adjusted correlations and agreement between biomarkers and Brazilian Healthy Eating Index-Revised components.

Biomarkers/BHEI-R Components	Crude Correlation *	*p*	Partial Correlation ^†^	*p*	Agreement in Same Quartile (%)	Misclassification in Opposite Quartile (%)
*LA*						
Total vegetables and legumes	0.24	0.002	0.25	0.002	34.2	9.3
Total fruits	0.23	0.004	0.23t	0.003	30.4	8.7
*ALA*						
Total vegetables and legumes	0.36	<0.001	0.36	<0.001	37.3	5.6
DGOV and legumes	0.22	0.005	0.25	0.001	32.9	6.8
Total fruits	0.26	0.001	0.30	<0.001	27.9	6.2
Meat, eggs and legumes	0.19	0.02	0.14	0.04	_	_
*EPA*						
Total vegetables and legumes	0.33	<0.001	0.27	0.001	34.2	7.4
Total fruits	0.29	<0.001	0.26	0.001	36.0	6.2
*DHA*						
Total vegetables and legumes	0.34	<0.001	0.34	<0.001	31.0	4.9
DGOV without legumes	0.18	0.03	0.16	0.04	25.5	8.1
Total fruits	0.29	<0.001	0.29	<0.001	33.5	6.2
Meat, eggs and legumes	0.16	0.04	0.19	0.02	_	_
*ARA*						
Total vegetables and legumes	0.32	<0.001	0.31	<0.001	27.9	5.6
Total fruits	0.29	<0.001	0.30	<0.001	34.2	6.8
*β-carotene*						
Total vegetables and legumes	0.19	0.016	0.25 ^‡^	0.002	35.7	9.5
DGOV and legumes	0.16	0.047	0.25 ^‡^	0.02	27.4	11.4
DGOV without legumes	0.27	0.001	0.21 ^‡^	0.01	29.3	6.4
Total fruits	0.24	0.002	0.19 ^‡^	0.02	33.1	8.9
Whole fruits	0.36	<0.001	0.25 ^‡^	0.002	38.2	7.0
*Retinol*						
DGOV and legumes	0.17	0.03	0.18 ^‡^	0.01	34.4	10.0
DGOV without legumes	0.21	0.007	0.20 ^‡^	0.02	32.5	9.4
Milk and dairy	0.18	0.02	0.19 ^‡^	0.02	26.9	10.0
*Riboflavin*						
Whole fruits	0.20	0.02	0.17	0.04	28.5	11.3
*Pyridoxal*						
Milk and dairy	0.16	0.042	0.21	0.007	27.8	10.1
*5-MTHF*						
Whole grains	0.21	0.04	0.27	0.01	_	_
*SAH*						
DGOV without legumes	0.18	0.004	0.21	0.006	28.4	5.9
*Creatine*						
Total vegetables and legumes	0.27	0.01	0.31	0.003	38.6	4.5
DGOV and legumes	0.33	0.002	0.37	0.001	38.6	4.5
Meat, eggs and legumes	0.42	0.000	0.34	0.001	_	_

Abbreviations: 5-MTHF—5 methyl tetrahydrofolate; ALA—α-linolenic acid; ARA—arachidonic fatty acid; DGOV—dark green and orange vegetables DHA—docosahexaenoic fatty acid; EPA—eicosapentaenoic fatty acid; LA—linoleic acid; SAH—*S*-adenosyl-homocysteine. * Spearman’s correlation. ^†^ Adjusted for age, sex and body mass index and ^‡^ for plasma cholesterol. Agreement analyses were not possible for pairs with dash “-”.
